# The Role of Neurokinin-1 Receptor in the Microenvironment of Inflammation and Cancer

**DOI:** 10.1100/2012/381434

**Published:** 2012-04-01

**Authors:** Marisa Rosso, Miguel Muñoz, Michael Berger

**Affiliations:** ^1^Research Laboratory on Neuropeptides, Hospital Infantil Universitario Virgen del Rocío, Avenida Manuel Siurot s/n, 41013 Seville, Spain; ^2^Department of Pediatric Infectious Diseases and Immunology, Hospital Infantil Universitario Virgen del Rocío, Avenida Manuel Siurot s/n, 41013 Seville, Spain; ^3^Department of Pediatric Surgery, Dr. von Hauner Children's Hospital, Ludwig-Maximilians-University Munich, Lindwurmstrasse 4, 80337 Munich, Germany

## Abstract

The recent years have witnessed an exponential increase in cancer research, leading to a considerable investment in the field. However, with few exceptions, this effort has not yet translated into a better overall prognosis for patients with cancer, and the search for new drug targets continues. After binding to the specific neurokinin-1 (NK-1) receptor, the peptide substance P (SP), which is widely distributed in both the central and peripheral nervous systems, triggers a wide variety of functions. Antagonists against the NK-1 receptor are safe clinical drugs that are known to have anti-inflammatory, analgesic, anxiolytic, antidepressant, and antiemetic effects. Recently, it has become apparent that SP can induce tumor cell proliferation, angiogenesis, and migration via the NK-1 receptor, and that the SP/NK-1 receptor complex is an integral part of the microenvironment of inflammation and cancer. Therefore, the use of NK-1 receptor antagonists as a novel and promising approach for treating patients with cancer is currently under intense investigation. In this paper, we evaluate the recent scientific developments regarding this receptor system, its role in the microenvironment of inflammation and cancer, and its potentials and pitfalls for the usage as part of modern anticancer strategies.

## 1. Introduction

Cancer research in general has seen an endorsed and exponential increase in the recent years and extensive financial venture and manpower have been invested in the field. Nonetheless, with the exception of very few specialized areas such as haematological cancers and perhaps certain skin cancers, this determination has not yet converted into a better prospect for cancer patients in general. Morgan et al. recently published an analysis on the contribution of chemotherapy in adult malignancies with respect to 5-year survival [1]. In this analysis, for 22 adult malignancies treated between 1990 and 2004, the overall contribution of curative and adjuvant cytotoxic chemotherapy was estimated to be close to 2%.

Nevertheless, there are several encouraging areas of investigation in cancer research. One field of particular interest is the identification of the tumor microenvironment as an essential part of tumor survival [2–4]. In better understanding the biology of the tumors and the microenvironment in which they flourish, researchers hope to identify novel molecular targets for the therapeutical inhibition of tumor growth. The neurokinin-1 (NK-1) receptor has recently been discovered to play an integral role in the maintenance of a favourable tumor microenvironment. Its pharmaceutical blockage robustly inhibits tumor growth of various tumors, making it an attractive anticancer target [5].

The NK-1 receptor is a tachykinin receptor. Three mammalian tachykinin receptors subtypes have been characterized, *TACR1* (NK-1 receptor), *TACR2,* and *TACR3*, which show preferential but not absolute selectivity for substance P (SP), neurokinin A (NK-A), and neurokinin B (NK-B), respectively [6, 7]. Tachykinin receptors are expressed by many different cell types and respond to tachykinins in a cell type-specific manner. The tachykinin receptors belong to the G protein-coupled receptors (GPCRs). GPCRs comprise a large family of membrane receptors involved in signal transduction. These receptors are linked to a variety of physiological and biological processes such as regulation of neurotransmission, pain, inflammation, cell growth and differentiation, and oncogenesis, among others.

Tachykinins are signalling molecules that bind to specific GPCRs on target cells. The NK-1 receptor is functionally coupled with G protein, and by the usage of several techniques including autoradiography, expression of mRNA encoding the NK-1 receptor protein, and immunocytochemistry, a widespread distribution of the NK-1 receptor has been observed in both the mammalian central nervous system as well as peripheral tissues [8–12].

SP and the subsequent activation of the NK-1 receptor lead to phosphoinositide hydrolysis [13], calcium mobilization [14], and mitogen-activated protein kinase (MAPK) activation [15, 16]. The activation of the NK-1 receptor was found to be involved in a myriad of processes related to oncogenesis such as mitogenesis, angiogenesis, cell migration, and metastasis. Therefore, SP and NK-1 receptor interaction strongly influence the tumor microenvironment.

In this respect, it has been demonstrated that SP acts through the NK-1 receptor as a mitogen in several human cancer cell lines, including astrocytoma, melanoma, neuroblastoma, glioma, retinoblastoma as well as pancreatic, larynx, colon, and gastric carcinoma, leukemia, and breast cancer [17–25].

Given the relevance of SP/NK-1 signaling in cancer, substantial efforts have been made to develop therapeutic inhibitors of the NK-1 receptor [3, 5]. Importantly, NK-1 receptor antagonists are considered to have promising antitumoral properties while having few side effects. In this paper, we review the recent scientific developments regarding the NK-1 receptor and its biological influence in tumor microenvironment and inflammation. The SP/NK-1 signaling cascade has great potential for pharmaceutical tumor targeting, and understanding of the role of NK-1 and its ligand SP might help to design modern, specific anticancer tumor strategies.

## 2. Interaction of Substance P and the Tachykinin Receptor

Human tachykinins are encoded by the *TAC1*, *TAC3, *and *TAC4 *genes, which express SP, NK-A, and NK-B peptides that interact with the tachykinin receptors NK-1, NK-2, and NK-3, respectively [17]. Hemokinin-1 (HK-1) is the most recent addition to the tachykinin family and demonstrates a similar binding affinity and preference for the NK-1 receptor as others tachykinins. After binding to the NK-1 receptor, SP regulates a variety of biological functions [26–29]. It has been implicated in the regulation of the cardiovascular system, in the dilatation of the arterial vascular system, in neuronal survival and degeneration, in the regulation of respiratory mechanisms, in sensory perception, in movement control, in gastric motility, in salivation, in micturition [30–32], in pain, and in depression [33–35]. The neuropeptide SP has also been implicated the process of wound healing where it facilitates neurogenic inflammation [36, 37].

Both SP and the NK-1 receptor are overexpressed in several cancers including breast, ovarian, prostate, pancreas, leukemia, and thyroid cancer as well as glioblastoma, among others [5, 17, 18]. In breast cancer, the involvement of SP and its receptor has been widely described for the acquisition of oncogenic properties and in the formation of bone marrow metastasis [38–41]. Activation of NK-1 receptor by SP results in the activation of PI3K, the NF-kB pathway, and (MAPKs). It has also been observed that SP can induce signal transduction through the NK-1 receptor by transactivating additional receptors. These receptors include receptors with tyrosine kinase activity, such as, for example, receptors belonging to the ErbB family, including EGFR and Her2. This effect was described in several cell types [42–44]. Furthermore, SP has been implicated in angiogenesis by inducing the formation of capillary-like structures from endothelial cells placed on a Matrigel matrix [45]. Activation of NK-1 receptors by SP can directly stimulate the process of neovascularization, probably through the induction of endothelial cell proliferation. SP-enhanced angiogenesis most likely results from a direct action on microvascular NK-1 receptors [46].

**Figure 1:**
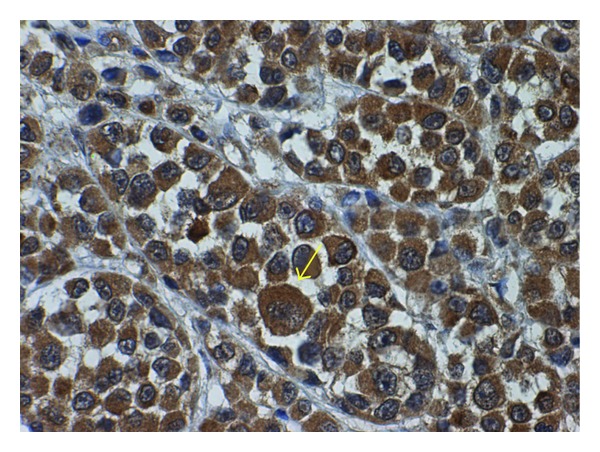
Immunohistochemical staining of the NK-1 receptor in a primary invasive malignant melanoma. A high expression of NK-1 receptor (arrow) was observed in the cytoplasm of the tumor cells. Cell nuclei were counterstained with hematoxylin (x 40).

In general, overexpression of immunoreactive NK-1 receptors is clearly confined to the plasma membrane of tumor cells although immunoreactivity at times shows a diffuse cytoplasmic staining [25]. In this regard, NK-1 receptor visualization with immunohistochemical methods has facilitated the identification of tumors with a sufficient degree of receptor overexpression for diagnostic or therapeutic intervention using NK-1 receptor antagonists [47] (Figure 1). 

Previous reports have shown that different isoforms of the NK-1 receptor can be found in both human [48–51] and rat tissues [51, 52], the isoform sizes of the latter ranging from 46 kDa to 54 kDa. For instance, in human lymphocytes, a glycosylated NK-1 receptor of 58 kDa is found [49], whereas this form and another two forms of 38 kDa and 33 kDa are also present in IM9 lymphoblasts [50, 51]. In the last few years, in several human tumor cell lines, the presence of four more abundant isoforms (75, 54–58, 46, and 33–38 kDa) of the NK-1 receptor has been described. Thus, in neuroblastoma SKN-BE(2), a major 54 kDa band is observed, whereas in the glioma GAMG cell line, an additional band of 33–38 kDa is seen [20]. In IMR-32 neuroblastoma cell lines, KELLY neuroblastoma cells, and T-ALL BE-13 cells, a band of 33 kDa and a major 54–58 kDa band are observed, and in IMR-32 neuroblastoma cells and B-ALL SD-1 cells, an additional isoform of about 75 kDa has been detected [18, 53]. In the pancreatic carcinoma CAPAN-1 and PA-TU 8902 cell lines, bands of 75, 58, 46, and 34 kDa have been described [54, 55]. The presence of 75, 58, and 33 kDa isoforms of the NK-1 receptor in the human retinoblastoma WERI-Rb-1 and Y-79 cell lines has been reported [53]. Moreover, isoforms of 75, 58, 46, and 34 kDa were observed in the human larynx carcinoma HEp-2 cell line and the gastric adenocarcinoma 23132/87 cell line, whereas the colon adenocarcinoma SW-403 cell line expressed isoforms of 75, 58, and 34 kDa [23, 25]. It should be noted that tumor cell lines as different as human retinoblastoma, neuroblastoma, glioma, larynx carcinoma, gastric and colon adenocarcinoma, and pancreas carcinoma expressed the same isoforms of the NK-1 receptor. In the future, the functional roles of the different isoforms of the NK-1 receptor observed in human cancer cells must be investigated, since their functional roles are currently unknown.

It is important to consider not only the qualitative but also the quantitative expression of NK-1 receptors by tumor cells, since it has been demonstrated that NK-1 receptor expression is increased 25–36-fold in human pancreatic cancer cell lines in comparison with normal controls, and that tumor samples from patients with advanced tumor stages exhibit significantly higher NK-1 receptor levels [55]. Thus, the number of NK-1 receptors expressed in normal human cells is lower (e.g., human blood T-lymphocytes express 7,000–10,000 NK-1 receptors/cell) [56] than that expressed in human tumor cells (e.g., astrocytoma cells express 40,000 NK-1 receptors/cell) [57]. An increased percentage of NK-1 receptor expression has also been reported in astrocytoma and glioblastoma tumors possessing the most malignant phenotypes, and the expression of NK-1 receptors is believed to correlate with the degree of malignancy. For example, glioblastomas express more NK-1 receptors than astrocytomas [58]. Moreover, it has been described that primary astrocytoma/glioma tumors express more NK-1 receptors than do established astrocytoma/glioma cell lines in culture [15]. Finally, the expression of mRNA coding the NK-1 receptor is increased in malignant tissues but not in benign tissues (e.g., breast biopsies) [41].

### 2.1. NK-1 Receptor Activation and Signalling

**Figure 2:**
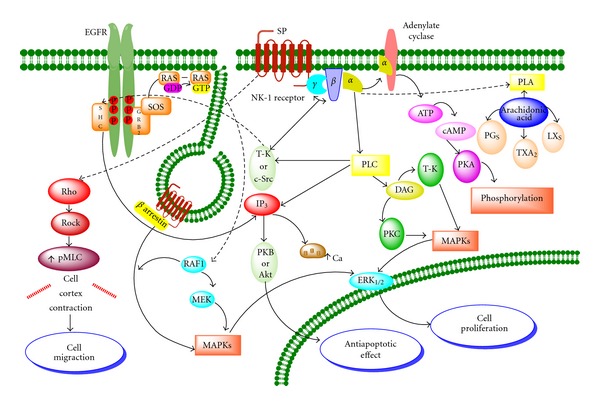
The downstream signaling pathways of the NK-1 receptor are shown. Activation of this receptor by SP leads to cell proliferation, antiapoptotic effect, and cell migration (see detailed description in text. Amplified from Muñoz et al. [66]).

SP preferentially binds to the G protein-coupled NK-1 receptor [59]. The coupling of GPCRs, also called serpentine receptors, to different G proteins is dictated by the receptor's amino acid sequence, particularly in the cytoplasmic loops and the carboxy-terminal tail of the receptor. This G complex consists of three different subunits: G*α* subunit that binds GDP/GTP, and G*β* and G*γ* subunits that form the complex G*βγ*. The activation of the receptor by an agonist induces the G*α* subunit to change GDP by GTP and its dissociation from the G*βγ* dimer, which has strong unions among its subunits G*β* and G*γ*. The subunits G*α* and G*βγ* (the kinase Src) then dissociate from the receptor and induce their own signalling cascade. G protein G*βγ* subunits recruit components of the ras-dependent cascade, such as shc, grb2, and src, leading to the activation of raf-1 and MAP kinase 1, a specific activator of ERK1y2 [60]. Once activated by its ligand SP, the NK1 receptor activates members of the MAPK cascade, including extracellular signal-regulated kinases 1 and 2 (ERK1/2) and p38MAPK. There are at least three different MAPKs: the extracellular signal-regulated kinase (ERKs), c-Jun NH2 terminal kinase (JNKs), and p38 MAPK. All have in common that they ultimately phosphorylate proteins related to the regulation of gene expression. In their activated state, the described pathways can lead to both growth and induction of apoptosis [61, 62]. The mechanisms by which these seemingly contradictory signals are conveyed is poorly understood although an emerging role for scaffolding protein complexes that determine the subcellular localization and consequent specificity of signaling proteins may provide an explanation [16, 63, 64]. The most commonly studied mechanism by which GPCRs activate MAPK is the release of G protein *βγ* subunits [63]. On the other hand, tyrosine kinase receptors (TKRs) comprise a family of cell surface proteins including most of the receptors for growth factors such as the ErbB family. Many TKRs share signalling pathways, and the biological responses specific to each receptor seem to depend more on the cell context than on any other factor. The ErbB family of receptors is composed of four members: EGFR (Her1), Her2, Her3, and Her4. The activation of an ErbB receptor by its ligands (with the exception of Her2, which lacks a ligand-binding domain) induces receptor dimerization, the activation of its intrinsic tyrosine kinase activity, and the transphosphorylation of the tyrosine residues present in its C-terminal domain of EGFR can be then recognized by various effector molecules that are responsible for spreading the signal as PLC*γ* and c-Src. The phosphorylation of these receptors, such as EGFR, also induces the tyrosine phosphorylation of the adapter protein SHC and the association of SHC with Grb2. Grb2 is constitutively associated with Sos1, a molecule responsible for catalyzing the exchange of GDP for GTP in Ras. Ras is a GTP-binding protein, which is anchored in the cell membrane and that once activated stimulates the above-mentioned MAPKs. These include MAPKKK, also known as Raf, which phosphorylates MAPKK, also known as MEK, which in turn is a component of the MAPK signaling pathway (Figure 2). Regarding Her2, the formation of a Src-Her2 heterocomplex has been described in breast cancer cell lines and in human tumors although the exact residue phosphorylated in Her2 remains unknown [65]. 

### 2.2. The SP/NK-1 Receptor Complex

It has been reported that upon exposure to the agonist SP, this peptide and its receptor are internalized into early endosomes within minutes of binding [67–70], that the loss of NK-1 receptors from the cell membrane is blocked by the inhibition of clathrin-mediated endocytosis, and that the recovery of NK-1 receptor expression at membrane level requires endosomal acidification but not novel protein synthesis. Internalized SP is intact in early endosomes but is slowly degraded in perinuclear vesicles [69]. Thus, SP induces a clathrin-dependent internalization of the NK-1 receptor; the SP/NK-1 receptor complex dissociates in acidified endosomes, and SP is then degraded, whereas the NK-1 receptor recycles to the cell surface [69]. *β*-arrestin, originally thought only to mediate receptor uncoupling and internalization, is required for activation of ERK1/2 by a number of GPCRs [16, 64, 71, 72]. As described above, the involved MAPKs phosphorylate proteins that then alter gene expression [73] (Figure 2). *β*-arrestin forms a complex with the internalized receptor, raf-1, and ERK1/2, retaining the activated kinases in the cytosol [16]. Thus, scaffolding complexes can determine the subcellular location and specificity of ERK1/2 and thereby govern the mitogenic potential of a given signal. A different *β*-arrestin complex, containing the *β*2-adrenergic receptor (*β*2-AR) and the tyrosine kinase src, also leads to ERK1/2 activation [64], but this signalling pathway mediates a distinct set of cellular responses, possibly because of different subcellular localization of the activated kinases. SP induces the formation of a multiprotein complex containing NK-1 receptor, *β*-arrestin, src, and ERK1/2, which forms close to the plasma membrane. Once activated, ERK1/2 translocates to the nucleus to induce proliferation and to protect from apoptosis [16] (see Figure 2). 

Some studies have indicated that the stimulation of the NK-1 receptor located in human glioblastoma cells by SP increases the phosphorylation and the activity of Akt or the protein kinase B (EC 2.7.11.1), a serine-threonine protein kinase that becomes activated *via *phosphatidyl-3-kinase (PI3K). The activation of Akt suppresses apoptosis [74, 75] (Figure 2). This is an important finding, because in glioblastoma, the basal activity of Akt is linked to a poor prognosis [76].

Interestingly, it also has been observed that NK-1 expression was related to the tumor cell subtype, being higher in the luminal cell lines expressing Her2 (SKBR3, BT474, and MDA-MD-453) than in the basal-like cell lines (MDA-MB-468, MDA-MB-231, MCF10A, and MCF12A). Moreover, the proapoptotic effect of NK-1 inhibition (as judged by annexin V positivity) was greater in cells with higher levels of NK-1 expression, suggesting “oncogenic addiction” of these cells to NK-1 signaling [42]. 

## 3. Tumor Microenvironment and the NK-1 Receptor

Traditionally, in cancer research, scientists have focused on the cancer cell itself. Only recently the microenvironment of tumors has been identified as an integral part of tumor growth and survival. The microenvironment of tumors includes any given interaction of the tumor cell with its surroundings, whether these are molecular or cellular structures. Integral components of the microenvironment of tumors include but are by no means limited to epithelial cells, fibroblasts and other stromal cells, extracellular matrix, autocrine and paracrine cytokines and their receptors, cell-to-cell contact among tumor cells and/or immune cells, angiogenesis, residence of cancer stem cells (CSCs), and many others. The tumor microenvironment has been shown to restrict the effectiveness of the antitumor immune response by displaying a variety of immunosuppressive strategies, making many effective anticancer treatment strategies used to date futile.

### 3.1. Angiogenesis

Neovascularization or neoangiogenesis is a sequential process. Early endothelial proliferation is followed by new vessel formation and increased blood flow accompanied by maturation of endogenous neurovascular regulatory systems [77]. Importantly, neoangiogenesis, which is considered a hallmark of tumor development, has been associated with increased tissue innervation and expression of NK-1 receptors. It is also known that in a large majority of tumors, SP and NK-1 receptors are found in the intra- and peritumoral blood vessels [58], and that SP, a main mediator of neurogenic inflammation through the release of the peptide from peripheral nerve terminals, is involved in the growth of capillary vessels *in vivo *and in the proliferation of cultured endothelial cells *in vitro*. Moreover, it is known that the proliferation of endothelial cells by NK-1 receptor agonists increases in a concentration-dependent manner whereas the action of selective NK-2 and NK-3 receptor agonists has no significant effects on the proliferation of endothelial cells. These findings indicate that NK-1 receptor agonists (e.g., SP) can directly stimulate the process of neovascularization, probably through the induction of endothelial cell proliferation, and that SP-enhanced angiogenesis results from a direct action on microvascular NK-1 receptors [46]. Thus, through such receptors found at high density in blood vessels, SP may strongly influence vascular structure and function inside and around tumors by increasing tumoral blood flow and by fostering stromal development, consequently influencing cell proliferation, survival, and migration [58].

### 3.2. Metastasis

One model for the development of metastasis is based on the finding that GPCRs regulate the migratory activity of tumor cells in a similar way as the recruitment and homing of leucocytes. Furthermore, ligands (e.g., neurotransmitters) to these receptors can induce directed chemotactic migration [78].

Migration is a prerequisite for invasion and metastasis and is dependent on signaling substances of the immune and neuroendocrine systems. The NK-1 receptor is an important regulator of motility in a variety of cells [79–83]. A recent study demonstrated that activation of the NK-1 receptor triggers complex and rapid cellular shape changes, including blebbing, in HEK293 cells. The cellular shape changes can be quantified using a cellular assay based on electrical impedance measurements in cellular monolayers. The NK-1 receptor-induced blebbing is not associated with apoptosis, and the main intracellular signaling mechanisms activated by the NK-1 receptor that are responsible for SP-induced cellular shape changes have been identified. Cell shape changes are dependent on Rho/Rock activation and are independent of phospholipase C activation, cytosolic calcium increase, and PKC activation [84] (see Figure 2).

The NK-1 receptor also plays an important role in breast cancer cell growth and in the integration of these cells into the bone marrow [41]. The NK-1 receptor is located on lipid rafts and caveolas, with its activity totally dependent on the microarchitecture of the cell membrane, as observed when cells are depleted of cholesterol. When the NK-1 receptor is activated by SP, the protein PKC is relocated on lipid rafts [85]. It appears that the NK-1 receptor can transactivate EGFR in glioblastoma cells, a process that probably depends on the colocation of both receptors in membrane microdomains [43]. In fact, it has been observed via confocal microscopy that EGFR colocalizes with lipid rafts and that the integrity of these lipid rafts is necessary for induction of chemotaxis by EGF [86].

### 3.3. Haematopoiesis and Cancer Stem Cells

Understanding the earliest development in cancer formation and progression is essential for introducing new anticancer targets. There are currently two hypotheses discussed that describe the natural progression of tumor cells. On the one hand, it is believed that any cell within a cancerous tumor has the ability to form and maintain the tumor mass. This is called the stochastic hypothesis [87]. The second, the so-called hierarchical hypothesis, suggests the existence of specialized CSCs [87–90]. CSCs are a rare population of cancer cells exhibiting stem cell properties, such as self-renewal, differentiation, and tissue restoration. These stem cells are in a particular niche, have a defined progenitor phenotype, higher resistance to chemotherapy and radiotherapy, and are also capable of invading and migrating to other tissues [87]. Therefore, beside the initiation of the primary tumor, CSCs have been associated with metastasis formation and cancer relapse [90].

Given the fact that the concept of CSCs has evolved very recently, it is not surprising that there exists to date so scarce literature regarding the expression and function of the SP/NK-1 receptor system in CSCs. Nevertheless, the crucial role of the SP/NK-1 receptor system in the development, maintenance, and spread of cancer mandates intense study of this receptor system in CSCs and related cells, such as metastatic CSCs (mCSCs). mCSCs are believed to be a specialized population within CSCs and have been proposed to be the essential seeds that initiate tumor metastasis [91]. Few data regarding this highly interesting scientific area do exist. For example, Li et al. studied the expression of SP and the NK-1 receptor in the established human stem cell line TF-1 and in primary stem cells derived from human placental cord blood (HPCB) and demonstrated that both SP and NK-1 are expressed in these cells [92]. We did not find literature analysing the SP/NK-1 receptor system in specialized CSCs. In recent reviews regarding CSCs, no mention of NK-1 or SP is made, supporting the need for further investigation in this matter [87–90].

In comparison, the role of SP and other tachykinins in physiological haematopoiesis is well established [93–95]. Additionally, tachykinins and their interaction with their receptors seem to be important in the neoplastic transformation of bone marrow, leading to the development of acute leukaemia and other haematological cancers [93, 96, 97]. HK-1 is known to have a crucial role in the regulation of bone marrow microenvironment, both physiologic and towards malignant transformation [98]. For example, in a tachykinin 4 gene knockout mouse (*TAC4−/−*), the early stages of B lymphocyte development are altered, leading to an increase of CD19(+)CD117(+)HSA(+)BP.1(−) “fraction B” pro-B cells in the bone marrow, whereas pre-B, immature, and mature B cells are unaffected. TAC4 encodes for the HK-1 receptor. These findings suggest an inhibitory role for HK-1 on developing B cells, and that it is part of the bone marrow microenvironment that supports and regulates the proliferation and differentiation of hematopoietic cells [96]. In another study, the presence of SP in B lymphocytes of hypoplastic bone marrow predicted neoplastic transformation [97]. Also, HK-1 enhances B-cell proliferation and antibody production [99], promotes survival of dendritic cells [100], enhances the proliferation of T-cell precursors, and increases the number of thymocytes [101] as well as decreases blood pressure [102].

Taken together, the described findings strongly suggest that SP and its interaction with the NK-1 receptor have a critical role in the tumor microenvironment. The SP/NK-1 receptor complex should be considered as a universal mitogen in NK-1 receptor-expressing tumor cell types, as it governs not only the neoangiogenesis and growth of the tumoral mass, but also the peritumoral infiltration and metastasis. Obviously, this is especially relevant in those tumor cells and tumoral and peritumoral tissues in which the SP/NK-1 receptor system is highly overexpressed, the later of which can include inflammatory cells, fibroblasts, blood vessels, nerves, and ganglia, as described above. SP stimulates mitogenesis and exerts an antiapoptotic effect by activating NK-1 receptors in tumor cells mainly via four mechanisms: (1) through an autocrine mechanism, by which SP is secreted from primary tumors, (2) through a paracrine mechanism, by which SP is released from tumor cells acting on endothelial cells and on other surrounding cells in the tumor microenvironment, (3) through means of the peripheral nervous system, since SP is released from peripheral nerve terminals, and possibly, (4) through an endocrine mechanism, related to emotional behaviour, by which SP reaches the peripheral tumors through the blood stream [66].

## 4. Substance P and the NK-1 Receptor in Inflammation and Cancer

Several levels of evidence indicate that SP plays a role in regulating neurogenic inflammation and immune responses in both peripheral tissues and in the central nervous system, as well as in pain [59, 70]. It is known that SP is a potent vasodilator in several peripheral tissues, that terminal nerve endings containing SP are located close to blood vessels, that the release of SP in peripheral tissues reproduces many of the physiological changes seen in acute inflammation, for example, plasma extravasation, and that a dramatic upregulation in the expression of the NK-1 receptor occurs during painful chronic conditions [103–108].

The SP/NK-1 receptor system can modulate the immune function from sensory nerves via neurogenic inflammation. Moreover, it is known that activation of the SP/NK-1 receptor system produces alterations in the humoral [107] and cellular, normal [108, 109], and tumoral immune response [20–25, 53]. Also, it has been reported that tachykinins are capable of modifying the response of a variety of inflammatory cells, including mast cells, granulocytes, lymphocytes, monocytes, and macrophages, and that oedema formation is induced by lower concentrations of SP and is blocked by NK-1 receptor antagonists [77]. Finally, SP regulation of immune cell function could originate not only from neuronal sources such as sensory nerves and neurogenic inflammation, but also from nonneuronal elements, such as eosinophils and macrophages, in which the expression of both SP and the NK-1 receptor is upregulated during inflammation [110, 111].

In addition, an increase in the levels of SP has been described during intestinal inflammation, and a significant correlation between the degree of inflammation and the clinical status of the disease has been implied [112, 113]. These observations indicate that local release of SP (e.g., in lymph nodes) might be a factor contributing to the immune disorder underlying chronic inflammatory bowel disease, since the presence of specific SP receptors on human peripheral blood T lymphocytes has been demonstrated [114]. SP does not act solely as neurotransmitter but can also function as a proinflammatory cytokine participating in acute inflammation and activating proteins involved in cell proliferation.

Interestingly, there is data to support a pathognomonic connection between inflammation and cancer with regards to the SP/NK-1 receptor complex. One of the theories that explains how an inflammatory process could induce or promote cell transformation suggests that a cancer is generated by a “wound that does not heal” [115, 116], or in other words, that cancer could be originated from a tissue that has suffered an improper repair after an injury. The mechanism of tissue repair seems also to be mediated by TKRs transactivation and, in some cases, the activation of these receptors is essential for proper tissue repair. Therefore, an erroneous or prolonged repair process would create a source of continuous stimulation of different receptors that eventually could induce cellular transformation [42].

Chronic inflammation is well documented to correlate with an increased risk of developing cancer [117], since inflammation increases both mitogenesis and mutagenesis [118]. It is also known that a dividing cell has a greater risk of mutation than a quiescent cell [119]. Cell division allows adducts to convert to mutations. The time interval for DNA repair during cell division is short, and endogenous or exogenous damage is therefore generally increased if cells are proliferating. Moreover, it is well established that cancer arises in chronically inflamed tissue, and this is particularly notable in the gastrointestinal tract [120]. Inflammation may become chronic either because an inflammatory stimulus persists or because of dysregulation in the control mechanisms that normally turn the process off. It has been reported that many of the cells, cytokines, and additional processes such as leukocyte migration, dilatation of the local vasculature, and angiogenesis involved in inflammation are found in a variety of tumors.

Chronic inflammation caused by intestinal flora leading to inflammatory bowel disease such as ulcerative colitis and Crohn's disease is clearly linked to a higher incidence of colon cancer. Moreover, elevated levels of SP and upregulated NK-1 receptor expression have been reported in the rectum and colon of patients with inflammatory bowel disease [121]. A very important study regarding the correlation of inflammation and cancer with regards to the NK-1 receptor was recently published by Gillespie et al. [122]. In this study, archival formalin-fixed paraffin-embedded colonic tissue from patients with ulcerative colitis who developed carcinoma or high-grade dysplasia was examined for changes in expression of the NK-1 receptor. They found the levels of the NK-1 receptor to be increased by 40% in high-grade dysplasia and 80% in carcinoma compared with quiescent colitis, therefore concluding a functional role for the NK-1 receptor in malignant transformation in colitis-associated cancer.

Analogously, dietary intake of proinflammatory carcinogens has been associated with prostate cancer. Chronic inflammation resulting from esophageal reflux gives rise to gastroesophageal reflux disease (GERD) and Barrett's esophagus, which is proven to be linked to a higher incidence of cancer. Chronic *Helicobacter pylori* infection produces chronic inflammation and is a documented potential risk factor for stomach cancer [123]. It is also known that the risk of pancreatic cancer is significantly elevated in subjects with chronic pancreatitis and appears to be independent of sex, country, or type of pancreatitis [124]. Likewise, the upregulation of mRNA expression coding for the NK-1 receptor has been reported in chronic pancreatitis, with a strong relationship to the pain syndrome in these patients [125]. It is important to note again in this context that in the vast majority of tumors, investigated NK-1 receptors were found in intratumoral and peritumoral blood vessels [58], and that NK-1 receptors are highly expressed in blood vessels not only within the tumor mass but also in the peritumoral tissue [55]. All these observations suggest that chronic inflammation could enhance cancer through the SP/NK-1 receptor system, which is upregulated in the process of chronic inflammation. In this regard, it has been demonstrated that SP stimulates mitogenesis [18–21, 23–25, 53, 126, 127], and that NK-1 receptor expression is increased in cancer cell lines in comparison with normal controls [55]. In addition, the growth of the tumor mass, peritumor infiltration, and metastasis could be regulated by the SP/NK-1 receptor system overexpressed in tumor cells and in tumor and peritumor tissues [55].

Nevertheless, despite the overwhelming evidence stated between chronic inflammation and the formation of cancer, recent evidence also suggests that the specific activation of the immune system and thereby creation of an immune response might actually be highly beneficial in the treatment of cancer [128, 129]. In this promising concept called immunostimulation, regulatory dendritic cells and other cells of the innate immunity are activated artificially via so called pattern recognition receptors (PRRs) [130, 131]. This highly conserved family of receptors is widely expressed among different cells in all vertebrates and is an essential part of the innate immunity [128]. PRRs recognize molecular patterns, which are highly conserved motifs in the structure of organisms such as bacteria, virus, and protozoa. Activation of PRRs generally signals danger and leads to a complex immune reaction [128, 131].

Members of the toll-like receptor (TLR) family are considered the most intensely studied PRRs. So far, 13 receptors localized on different immune cells have been described, each unique in its ability to recognize molecular patterns from foreign organism as part of the natural immune defense mechanism. For example, TLR4 binds lipopolysaccharide from Gram-negative bacteria, TLR7 and TLR8 bind specific motives in RNA of certain virus, and TLR9 binds CpG motifs of bacterial DNA. Immune response generally involves but is by no means limited to an INF*γ* response [128].

In recent years, it has become evident that ligands against PRRs can be used in order to stimulate the immune system and thereby enhance the natural ability of the human body to fight malignancy and virus infection [130, 131]. This has created hope that such molecules could have great antitumor potential and can be used as part of modern adjuvant anticancer strategies. In our previous publications, we described the activation of the innate immune system with small, single-stranded RNA molecules leading to IFN-*γ* production by NK cells and CD8-T-cells and enhanced antitumor activity of these cells against target cells [132–134].

Therefore, while chronic inflammation is proven to cause cancer on the one hand, it seems that specific, partial activation for a defined period of time potentially might have the exact opposite effect, and the latter could be used as part of anticancer strategies via ligands of specific PRRs.

Another important, related aspect is the ability of SP to modulate the immune response. In cancer patients, both adaptive and innate cell-mediated immunity failed to prevent the development of the primary tumor but may be extremely important in eradicating residual diseases and in controlling metastasis [135, 136]. The high incidence in tumor recurrence after major surgeries might be, in fact, due to immunosuppression. Among several aspects of surgery involved in immunosuppression, the local neurogenic proinflammatory response initiated by nociceptive afferent nerve endings may have a prominent role [137]. It involves local and spinal reflexes and promotes erythema and edema around the surgical wound by releasing numerous compounds, including SP [138, 139]. Tachykinins, especially SP, may contribute to the enhancement in systemic levels of proinflammatory cytokines (interleukin (IL-6 and IL-8)) and an increase in plasma level of type 2 cytokines and other factors interfering with the cellular immunity (IL-10, IL-1rA, soluble tumor necrosis factor-*α* receptor sTNF-*α*r, sIL-2r) observed after surgery [140–142].

## 5. Emotional Behaviour, NK-1 Receptor, and Cancer

Since the beginning of the 20th century, awareness has been raised regarding psychosocial factors being implicated in the incidence and progression of cancer. The effect of these factors on tissues is thought to be a reflection of the release of certain, specific hormonal transmitters from cells of the neuroendocrine system. We have previously reflected this fascinating aspect of cancer pathology in depth [143]. The concise overview presented here is intended to outline the importance of this concept when developing modern, future anticancer strategies.

Depression and cancer co-occur commonly. The prevalence of depression among cancer patients increases with disease severity and symptoms such as pain and fatigue. New evidence supports the notion that emotional distress and nonexpression of emotions may adversely affect the clinical course of cancer [143]. Past research on the relationship between depression and cancer risk provided somewhat inconsistent evidence, and recent studies have suggested that chronic depression is a greater risk factor than episodic depression. Moreover, there is evidence that providing psychosocial support reduces depression, anxiety, and pain and may increase the survival time of cancer patients [143]. In this sense, there is evidence to suggest that factors related to lifestyle, such as exposure to various forms of stressors, are associated with mammary tumorgenesis. The possible role of life-style factors in breast cancer is important because the mortality due to this disease is increasing in most countries and the development of curative therapies for breast cancer has not been forthcoming for years. The crucial factor affecting tumor growth is the interaction between stressors, an individual's personality, and the available psychosocial support, as well as the effect of their interaction on an individual's ability to cope with stress [137, 144, 145].

It must also be emphasized that there are other possible mediating mechanisms for the influence of psychological factors besides the classical psychoneuroimmunological (PNI) pathway, such as the above-mentioned neoangiogenesis as part of a favourable tumor microenvironment [146]. Several cytokines, including interferons, angiogenin, tumor necrosis factor, transforming growth factors and interleukin-8 (IL-8), regulate this process. Since some studies have demonstrated an effect of psychological factors on cytokine production, a more specific, albeit speculative, PNI hypothesis can be proposed for the influence of immunological factors on tumor growth [147]. It has been reported that psychotropic drugs modify the expression of the genes encoding the synthesis of tachykinins and the expression of the NK-1 receptor in selected brain areas [148–150]. It is known that chronic treatment with antidepressant drugs produces a decrease in the concentrations of SP in several areas of the central nervous system, including the striatum, substantia nigra, and the amygdala. These findings suggest that a reduction in SP levels in certain brain regions could contribute to a common therapeutic effect of antidepressant drugs in affective disorders [137, 151].

SP and NK-1 receptors are present in the limbic system, including the hypothalamus and the amygdala. In addition, SP may be involved in the integration of emotional responses to stress, suggesting the possibility that the pathogenesis of depression could be due to an alteration of the SP/NK-1 receptor system [34]. In fact, in depression, an increase in the production of SP has been observed. In addition to the involvement of SP in depression, the NK-1 receptor antagonist L-733,060 has been used as an antidepressive agent [34, 35], and it has been demonstrated that this antagonist has antitumor activity against human glioma, neuroblastoma, melanomas, retinoblastoma, and pancreatic carcinoma cell lines [18, 20, 21, 23–25, 127]. Moreover, it is known that the NK-1 antagonist aprepitant (MK869), an agent structurally unrelated to L-733,060 or L-732,138 with antidepressant activity, is as efficient as the antidepressant drug paroxetine in the treatment of depression. Aprepitant is well tolerated, and no statistically significant differences in the frequency of adverse events as compared to placebo administration have been observed [34].

These data suggest that depression could induce tumor cell proliferation by activating the SP/NK-1 receptor system and that treatment with NK-1 receptor antagonists could be useful not only in depression, but also for the treatment of tumor cells. Moreover, all the above data indicate that emotional behaviour, for example, depression, and cancer are related through alterations in the SP/NK-1 receptor system [43, 145, 152, 153]. Accordingly, through this receptor system, there could be an interrelationship between the progression of cancer and cerebral mechanisms. The NK-1 receptor expressed in the limbic system is similar to the NK-1 receptor expressed in human tumor cell lines, and SP, after binding to NK-1 receptors located in the limbic system, produces anxiety and depression, whereas after binding to the NK-1 receptor of human tumor cells, SP induces cell proliferation. Therefore, the NK-1 receptor antagonists in the limbic system exert antidepressant and anxiolytic actions, while the NK-1 receptor antagonists in human tumor cell lines have antitumor action.

On the other hand, the cytokine response, not only to surgery but also to stress, is intertwined with the neuroendocrine response. During the perioperative period and following psychological stress and anxiety, neuropeptides including tachykinins [154] are released systemically as well as locally by nerve endings that are believed to form “synapses” with leukocytes [141, 148, 155]. Moreover, it has been reported that regional anesthesia and analgesia may help to attenuate the surgical stress response by inhibiting the release of SP from nerve fibers. In this sense, SP could be one of the perioperative factors, as suggested by others [152, 156], which increase tumor growth and spreading. These questions require future investigation for confirmation, but it leads us to believe that it is quite possible that, as in pain and in neurogenic inflammation, in tumor progression, in angiogenesis, and in tumor cell migration, the activation of the NK-1 receptors by SP plays an important role. Hence, the SP/NK-1 receptor system, after being blocked with NK-1 receptor antagonists or regional anesthesia, could be involved in the recurrence of cancers overexpressing NK-1 receptors [157].

## 6. NK-1 Receptor Antagonists

Substantial efforts have been made to develop therapeutic inhibitors against NK-1 receptors in the last decade. Small molecules as antagonists of the NK-1 receptor have represented an important opportunity to further exploit compounds that are active against this receptor as novel therapeutic agents [158]. The current NK-1 receptor antagonists have been recently examined in detail by Muñoz et al. [66].

In general, distinction is made between peptide antagonists and nonpeptide antagonists. With regards to peptide antagonists, most work carried out on the design and preparation of antagonists of the NK-1 receptor has focused on the introduction of D-amino acids [159]. However, their affinity is several orders of magnitude lower than that of natural agonists, and the metabolic instability of peptide NK-1 receptor antagonists and their inability to gain access to the central nervous system through the blood-brain barrier limits their usefulness for *in vivo *studies. In addition, after administration in the central nervous system, these substances generally suffer from a number of drawbacks, such as poor potency and an inability to discriminate between tachykinin receptors, partial residual agonist activity, degranulation of mast cells, and neurotoxicity [160, 161].

**Figure 3:**
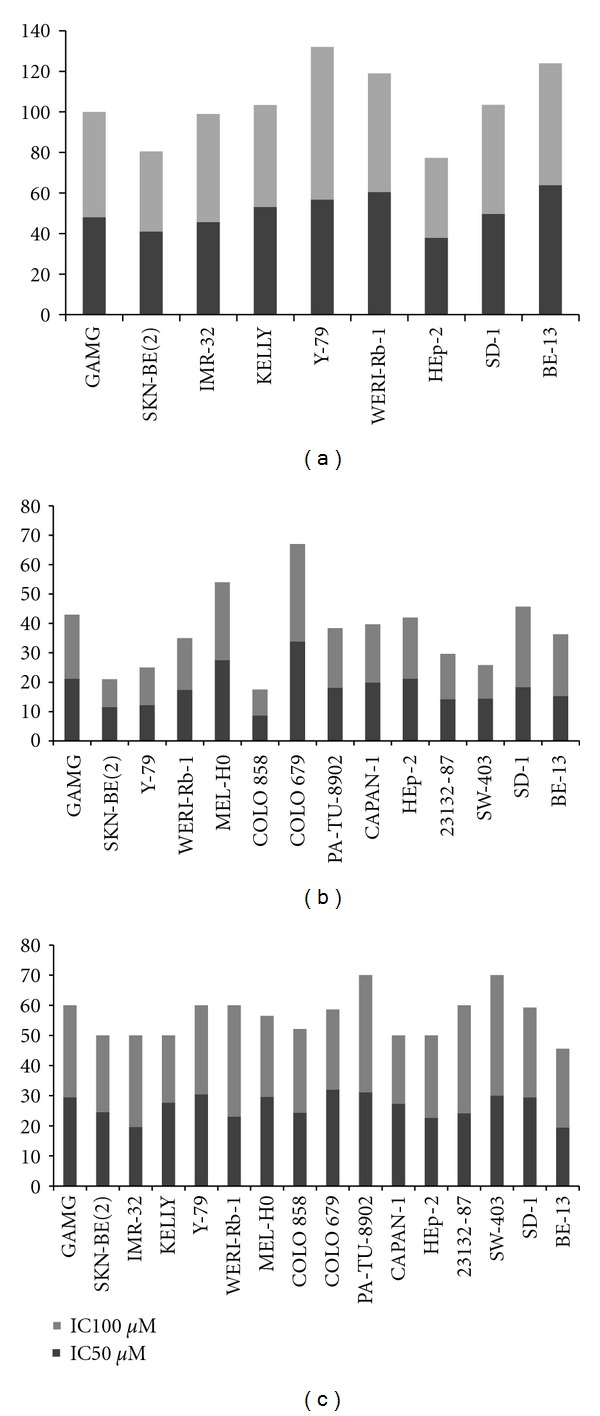
Cytotoxicity in human cell lines induced by NK-1 receptor antagonists. An up-to-date outline of our so far published results is shown for the antitumor effect of NK-1 receptor antagonists (a) L-732,138, (b) L-733,060, and (c) aprepitant in a variety of human cell lines of different tissues including glioma, neuroblastoma, retinoblastoma, melanoma, carcinoma of pancreas, larynx, stomach, and colon. Concentrations of the antagonists are shown in *μ*Molar for each cell line corresponding to 50 and 100 percent inhibition by cytotoxicity assay.

**Figure 4:**
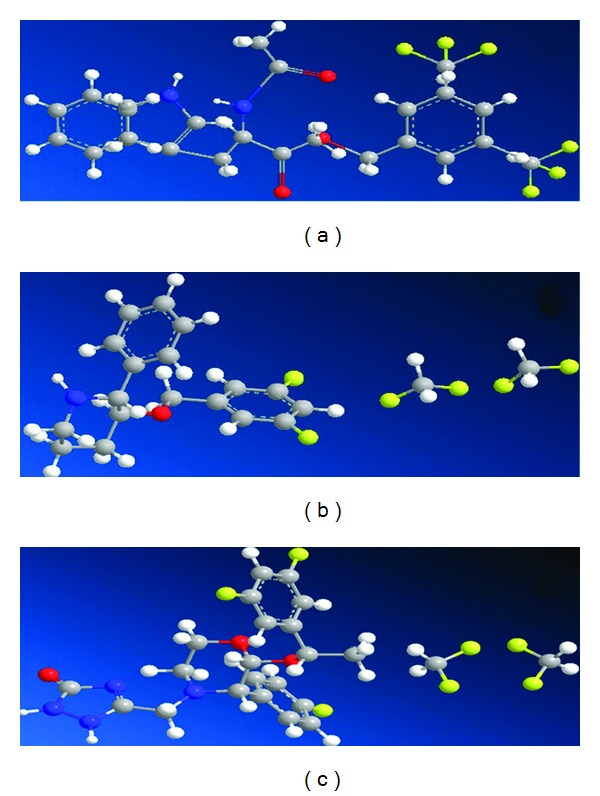
The chemical structure is shown for L-732,138 (a), L-733,060 (b), and aprepitant (c). Hydrogen elements are symbolized in white, carbon in grey, nitrogen in blue, oxygen in red, and fluorine in yellow colors.

The pitfalls encountered with the development and use of peptide antagonists against the NK-1 receptor lead to the search for alternative receptor antagonists. Different research groups searched for NK-1 receptor antagonists by screening a wide variety of chemical collections. Eastman Kodak and Sterling Winthrop initially proposed steroid compounds as tachykinin NK-1 receptor antagonists, but they had insufficient affinity for the NK-1 receptor [160, 162]. Moreover, these compounds proved to have significant toxicity. Although many derivatives of steroid compounds have been synthesized, their biological activity has not improved. Rhone-Poulnec proposed the compound RP-67,580, which showed a high affinity for the NK-1 receptor in rats and mice, but not in humans [160, 162]. Pfizer proposed a benzylamino quinuclidine structure called CP-96,345 [163]. It was a rather simple structure composed of a rigid quinuclidine scaffold containing a basic nitrogen atom, a benzhydryl moiety, and an o-methoxybenzylamine group. This compound showed high affinity for the NK-1 receptor but also interacted with Ca^2+^-binding sites, which caused a number of systemic effects unrelated to the blocking of the NK-1 receptor. The compound CP-99,994 was synthesized by replacing the quinuclidine ring by a piperidine ring and the benzhydryl moiety by a benzyl group. CP-99,994 showed a high affinity for the human NK-1 receptor, and several structure-activity studies were carried out in order to identify the structural requirements vital to increase its affinity for the NK-1 receptor. These studies reached a phase II clinical trial, but no further studies were carried out owing to its poor bioavailability. Pfizer obtained other related NK-1 receptor antagonists, for example, CJ-11,974, also called ezlopitant; it is a close analogue of CP-96,345 and contains an isopropyl group on the methoxybenzyl ring. It was developed up to phase II clinical trials for chemotherapy-induced emesis. CP-122,721 is a CP-99,994 analogue that contains a trifluoromethoxy group in the o-methoxybenzyl ring. It reached a phase II trial for the treatment of depression, emesis, and inflammatory diseases but ultimately failed. In 1993, Merck initiated studies on NK-1 receptor antagonists, based on both CP-96,345 and CP-99,994. L-733,060 is one of the compounds developed from CP-99,994 (Figure 4). It is a 3, 5-bistrifluoromethyl benzylether piperidine [164]. It is known that the administration of L-733,060 produces analgesia [33] and antidepressive effects [34, 35]. The compound has been suggested for the treatment of anxiety and mood disorders [165] and in inflammatory liver disease, most likely owing to its ability to inhibit the effects of SP [166]. In addition, it has been reported by our group that L-733,060 acts as an antitumor agent in several human tumor cell lines [18, 20, 21, 23–25, 127] (see Figure 3).

Further changes in the chemical structure of NK-1 receptor antagonists have created certain advantages. A morpholine nucleus that was introduced in L-742,694 was found to enhance NK-1 receptor-binding affinity [167]. This nucleus was kept in further modifications. In order to prevent possible metabolic deactivation, several refinements, such as methylation on the C alpha of the benzyl ring and fluorination on the phenyl ring, were carried out. These changes afforded the compound MK-869, which showed high affinity for the NK-1 receptor. MK-869 is also called aprepitant (Figure 4) and is used for the treatment of pain, migraine, emesis, and psychiatric disorders. These studies led the Food and Drug Administration (FDA) to approve the drug Emend, which is indicated for chemotherapy-induced nausea and vomiting and is available for oral use [168]. A water-soluble phosphoryl prodrug for intravenous use, called fosaprepitant, is also available and is marketed as Ivemend [169]. It seems that aprepitant is effective for the treatment of depression [34, 35] and cancer [170, 171].

## 7. Effects of NK-1 Receptors Antagonists

### 7.1. Antitumor Effects of the NK-1 Receptor Antagonists

In recent years, it has been demonstrated that L-733,060 displays antitumor activity against a large variety of cancer cell lines, including human SKN-BE(2) neuroblastoma, GAMG glioma, COLO 679, COLO 858, and MEL HO melanoma, WERI-Rb-1 and Y-79 retinoblastoma, and CAPAN-1 and PA-TU 8902 pancreatic, HEp-2 larynx, 23132/87 gastric, and SW-403 colon carcinoma cell lines and T-ALL BE-13 and B-ALL SD-1 leukemia cell lines [18, 20, 21, 23–25, 127] (see Figure 3).

Another NK-1 receptor antagonist, L-732,138, also elicits antitumor action against several human tumor cell lines (see Figure 3). Analysing the mechanisms for the observed antitumoral effect, it was demonstrated that the antitumor action is due to specific binding of the NK-1 receptor antagonists to the human NK-1 receptor. Thus, exogenous SP-induced cell proliferation was partially reversed by the administration of L-733,060 or L-732,138. This indicates that the antitumor action is specific and related to the ability of these antagonists to block the NK-1 receptors expressed by these tumor cell lines. It has also been demonstrated, as previously indicated, that the NK-1 receptor antagonist L-733,060 inhibits the metastatic progression of NK-1 receptor-expressing cancer cells [18, 19, 21–23, 25, 53, 172]. Furthermore, this antagonist binds specifically to the human NK-1 receptor-expressing cells in human breast carcinoma [153]. The growth inhibition observed in the above-reported human cancer cell types using the NK-1 receptor antagonists L-733,060 or L-732,138 is in agreement with the findings of other studies, in which the use of SP antagonists other than L-733,060/L-732,138 inhibited the growth of small cell lung cancer and the U373 MG glioma cell line [173, 174]. Moreover, these data are also in agreement with a recent study, in which the NK-1 receptor antagonist aprepitant was used. This drug is a broad spectrum antitumor drug which acts *in vitro *against neuroblastoma, glioma, retinoblastoma, and pancreatic, larynx, gastric, colon carcinoma, and leukaemia cell lines [18, 171] (see Figure 3).

As illustrated in Figure 3, L-733,060, L-732,138, and aprepitant induced growth inhibition by apoptosis in human A-204 rhabdomyosarcoma, in SKN-BE (2), KELLY, and IMR-32 neuroblastoma, in WERI-Rb-1 and Y-79 retinoblastoma, and in HEp-2 larynx, 23132/87 gastric, SW-403 colon carcinoma, and T-ALL BE-13 and B-ALL SD-1 cell lines [18, 22, 23, 25, 53, 54, 149, 175]. This is in agreement with other reports, since it is known that SP antagonists other than that mentioned above induce apoptosis in lung cancer and cause a concentration-dependent loss of cell viability [176].

It seems that the antitumor activity of the NK-1 receptor antagonists is a specific action and not a general toxic effect [5]. When interpreting these findings, one must bear in mind several important concepts. First, all the mentioned cell lines in which a robust antiproliferative effect can be induced by NK-1 receptor antagonists highly express NK-1 receptors. Further, the antitumor action of NK-1 receptor antagonists is dose dependent.

The NK-1 receptor antagonists L-733,060, L-732,138, and aprepitant are designed molecules that bind specifically to this receptor, with a Ki value of 0.8 mM in the case of L-733,060, of 2.3 nM for L-732,138, and of 0.2 nM for aprepitant. Thus, molecules that have structural similarities with NK-1 antagonists such as piperidine (similar to L-733,060), L-tryptophan (similar to L-732,138), and morpholine (similar to aprepitant, see Figure 4) exert the same antitumor action only by having in common their specificity for the NK-1 receptor. The blockade of NK-1 receptors in the above-mentioned human cell lines by L-733,060, L-732,138, or aprepitant was shown to inhibit both DNA synthesis and cell proliferation through the MAPK pathway [15] and additionally was found to inhibit the formation of a *β*-arrestin-containing complex that allows nuclear translocation of ERK1/2 and a decrease in the basal activity and expression of Her2 and EGFR inhibiting proliferation and inducing apoptosis [16, 177].

The foregoing data suggest that the antiproliferative action of the NK-1 receptor antagonists L-733,060, L-732,138, and aprepitant, once bound to the NK-1 receptors located in the tumor cells, most likely involves the interaction with a signal transduction pathway for apoptosis. It should be noted that in cell lines as different as human neuroblastoma, glioma, retinoblastoma, larynx carcinoma, and leukaemia the same NK-1 receptor antagonist (L-733,060,L-732,138, or aprepitant) elicits growth inhibition. This suggests the possibility of a common mechanism for cancer cell proliferation mediated by the SP/NK-1 receptor system. If this would result correctly, it would mean that NK-1 receptor antagonists could inhibit a large number of tumor cell types in which both SP and NK-1 receptors are expressed, and that they could be candidates for broad spectrum antineoplastic drugs [18, 22, 23, 25, 41, 53–55, 178–181].

It is also well known that SP activates malignant glial cells by inducing cytokine release and proliferation, both responses being relevant for tumor progression. The role of SP in supporting glioma progression *in vivo *is highlighted by the tumor growth inhibition induced by highly specific and selective human tachykinin NK-1 receptor antagonists (MEN 11,467 and MEN 11,149). The antitumor activity of MEN 11,467 was observed in both subcutaneous and intravenous treatments, and it was partially reversed by the concomitant administration of exogenous SP. These findings suggest a novel approach for the treatment of malignant gliomas with tachykinin NK-1 receptor antagonists [173]. Furthermore, the antitumor activity of NK-1 and NK-2 receptor antagonists has been demonstrated in nude mice, measuring growth inhibition of MDA-MB-231 tumor cells xenografted subcutaneously. A significant inhibition was found when the compounds were administered intravenously (5 mg/kg per day for 2 weeks). These results suggest that the *in vivo *activity of NK-1 and NK-2 antagonists may be a result of a cytostatic effect rather than a cytotoxic effect [173, 182]. Moreover, it has been reported that SP antagonists exert an antitumor action in a xenograft model of a human primary colon tumor cell line [183]. These findings are consistent with previous evidences showing that the inhibition of *TAC1 *expression by small interfering RNA in breast cancer cells shows a lack of tumorigenic phenotype and an inhibition of bone marrow metastasis in nude mice [39], while overexpression of NK-1 receptor leads to a transforming phenotype [184].

### 7.2. Inhibition of Angiogenesis and Migration of Tumor Cells

Neoangiogenesis, a hallmark of tumor development, has been associated with increased tissue innervation and the expression of NK-1 receptors [41]. As mentioned above, it is known that in a large majority of tumors investigated, SP and NK-1 receptors were found in the intra- and peritumor blood vessels [58]. A well-studied example of this high expression of NK-1 receptors in blood vessels not only within the tumor mass but also in the peritumorous tissue is pancreatic cancer [55]. It is known that SP analogue antagonists (SPA), synonymous of NK-1 receptor antagonists, block the endothelial proliferative action of SP, and that SPAs exert antitumorigenic activities. SPA, *in vitro, *inhibits multiple neuropeptide-induced Ca^2+^ mobilization, DNA synthesis, and growth in ductal pancreatic cancers. SPA also significantly attenuates the growth of HPAF (human ductal pancreatic adenocarcinoma)-II tumor xenografts in nude mice; markedly increases apoptosis, but only moderately decreases proliferation markers; markedly reduces tumor-associated angiogenesis in the HPAF-II xenografts *in vivo*; attenuates tumor growth in pancreatic cancer *via *a dual mechanism involving both antiproliferative and antiangiogenic actions [185]. These actions are likely to be related to the presence of NK-1 receptors in the tumor and in the endothelial cells [55, 58, 186]. Thus, the NK-1 receptor target could be used for the inhibition of angiogenesis, since it is known that SP stimulates vessel growth by enhancing endothelial cell proliferation and that, *in vivo, *angiogenesis is mimicked by selective NK-1 receptor agonists and inhibited by NK-1 receptor antagonists [46].

As mentioned above, development of metastasis can be explained in terms of the notion that GPCRs regulate the migratory activity of tumor cells. This means that ligands of these receptors could induce the migration of tumor cells and that migration could be regulated by signaling substances, including neurotransmitters. These could induce a metastatogenic tumor cell type by regulating gene expression and by increasing migratory activity. Accordingly, this activity can be prevented by neurotransmitter antagonists. It has also been reported that NK-1 receptors are present in the MDA-MB-468 breast cell line and that L-733,060 completely inhibited the SP-mediated increasing migratory activity [78]. Moreover, it has been described that in patients with breast cancer the risk of recurrence or metastasis is reduced four-fold during a 2.5-to-4-year follow-up period when surgery was associated with paravertebral anesthesia [142, 149, 156]. It seems that this occurs because paravertebral anesthesia blocked SP-induced migration, invasion, and metastasis of the tumor cells. Taken together, tumor-cell migration is a prerequisite for invasion, and metastasis and is dependent on the signaling substances of the immune, nervous, and neuroendocrine systems. Thus, the SP/NK-1 receptor system could regulate the growth of the tumor mass, the peritumor infiltration, and the metastasis, since in tumor cells and in tumor and peritumor tissues, this receptor system is highly overexpressed [55, 58].

### 7.3. Additional Effects

A variety of additional effects have been observed for NK-1 receptor antagonists. Recently, an *in vitro *study has demonstrated that L-733,060, combined with vinblastine or microtubule perturbing agents, is synergistic for the growth inhibition of the NK-1 receptor-possessing cancer cell lines (T98G, U87, Hela, T24, and MDA-MB-231), but not for normal lung IMR-90 fibroblast cells. This indicates that this combination is more potent against the NK-1 receptor overexpressing cancer cells and that the interaction between these molecules, the microtubule destabilizing agents (MDAs) and the NK-1 receptor antagonists, might be clinically useful (e.g., aprepitant has been approved by the FDA as an antiemetic for chemotherapy-induced emesis). These data demonstrate the usefulness of the MDAs and the NK-1 receptor antagonists to predict novel relationships between different classes of compounds for cancer chemotherapy [187].

Furthermore, it has been reported that the antineoplastic agent cyclophosphamide and the radiation can, respectively, produce neurogenic inflammation in the urinary bladder and in the gastrointestinal tract, such inflammation being mediated by NK-1 receptors. In this sense, the administration of NK-1 receptor antagonists in combination with certain cytostatic drugs and radiation therapy has a dual effect; it exerts a synergistic antitumor action, and it decreases cytostatic and radiation therapy side effects [188, 189]. It is also known that NK-1 receptor antagonists reduce plasma protein extravasations caused by antineoplastic drugs. In this sense, it seems that the neurogenic inflammation induced by cytostatics and radiation therapy is mediated by the release of SP from nerve terminals. Thus, after binding to NK-1 receptors located in the blood vessels, SP increases the permeability of these vessels, resulting in an extravasation of plasma proteins. This means that by blocking the NK-1 receptors with NK-1 receptor antagonists, the triggering of the inflammatory cascade could be aborted, and hence the lysis or apoptosis of neutrophils, enhanced by inflammatory mediators, is considerably decreased. These findings are very important, because cytostatics and radiation produce, first, an inflammation of the mucosa and, second, a breakdown of the mucosal barrier. These sites are a gateway for germs and elicit systemic infection, which is exacerbated by neutropenia secondary to the use of radiation and cytostatics. In contrast, the use of NK-1 receptor antagonists improves neurogenic inflammation, whether it is caused directly by radiation or indirectly by inflammatory mediators [188, 189].

NK-1 receptor antagonists have been observed to be effective analgesics. Intravenous administration of the NK-1 receptor antagonist L-733,060 to gerbils before an intraplantar injection of formalin caused a dose-dependent and complete inhibition of the late, but not the early, nociceptive response phase (paw licking). In contrast, the nonbrain penetrant quaternary ketone NK-1 receptor antagonist L-743,310 did not attenuate the response to formalin, indicating that the antinociceptive effect due to the blockade of NK-1 receptors by L-733,060 is centrally mediated. These data suggest that NK-1 receptor antagonists can be used as centrally acting analgesics [28, 33].

One additional effect of NK-1 receptor antagonists is of particular interest in this context. Due to their interaction with NK-1 receptors expressed in the central nervous system, the antagonists seem to elicit both anxiolytic and antidepressive effects. Oral administration of the NK-1 receptor antagonist L-733,060 (10 mg/kg, approximately 30 *μ*M) or other NK-1 receptor antagonists produced anxiolytic-like effects in the gerbil-elevated plus maze. It is also known that aprepitant (0.01–3 mg/kg) is the most potent NK-1 antagonist producing anxiolytic-like effects. These data suggest that NK-1 receptor antagonists may have clinical usefulness in the treatment of a range of anxiety and mood disorders [165]. In a preclinical assay, oral administration of the NK-1 receptor antagonist aprepitant drug (3 mg/kg), L-733060 (10 mg/kg, 30 *μ*M), or other NK-1 receptor antagonists revealed the antidepressant potential of these substances [35]. In a placebo-controlled trial in patients with moderate-to-severe major depression, robust antidepressant effects of the drug aprepitant were consistently observed [34]. There is currently accumulating abundant literature and scientific evidence showing that the NK-1 receptor antagonists vofopitant, ezlopitant, CP-122,721, and the drug aprepitant control chemotherapy-induced nausea, postoperative nausea, and vomiting [168, 190, 191]. These considerations are of particular interest when treating not just cancer as an isolated entity of disease, but when treating a whole person who suffers from cancer.

Additional effects of NK-1 antagonists include a potent anti-inflammatory and hepatoprotective effect. Pretreatment of mice with the NK-1 receptor antagonists CP-96,345 or L-733,060 (20 mg/kg, approximately 60 *μ*M) protected mice from GalN/LPS-induced liver injury. Also, NK-1 receptor blockade reduced inflammatory liver damage (e.g., edema formation and neutrophil infiltration). These findings can be interpreted in a way that NK-1 receptor antagonists exert a hepatoprotective effect [166].

### 7.4. NK-1 Receptor Antagonists as a Clinical Drug

It is important to note that the action of NK-1 receptor antagonists is dose dependent. Furthermore, depending on the dose, NK-1 receptors of different localization can be targeted, bearing even greater pharmaceutical potential. The following concepts are currently accepted with regards to the dosage of NK-1 receptor antagonists [192]. At low doses, they act on the NK-1 receptors of the brain stem located in the glossopharyngeal nucleus, with an antiemetic action in situations of intractable vomiting due to cytostatics. If the dose is raised, the drug acts on NK-1 receptors at the central level in the limbic system (amygdala, hypothalamus, and areas related to emotional behaviour), producing an antidepressive and anxiolytic action. At higher doses, they act on the thalamus and other brain areas as a central analgesic. Still higher doses produce an anti-inflammatory action, and similar or slightly higher doses, depending on the tumor in question and its stage, elicit antitumoral activity by the inhibition of cellular proliferation, metastases, peritumor inflammatory reaction, and angiogenesis. When used at its highest doses, NK-1 receptor antagonists produce all the pharmacological effects described above [192].

As mentioned above, NK-1 receptor antagonists specifically bind the NK-1 receptor. Therefore, they act selectively against tumor cells, because these cells express more NK-1 receptors than nontumoral cells. Also, their action is longlasting, because they are not hydrolysed by peptidases. Of particular interest is that these drugs generally can be administered orally with sufficient therapeutic effect, and that they cross the blood-brain barrier [66].

Moreover, safety and tolerability of the NK-1 receptor antagonist aprepitant was demonstrated in a placebo-controlled trial in patients with moderate-to severe major depression. At a dose of 300 mg/day, aprepitant was well tolerated, and no statistically significant difference in the frequency of adverse events was observed as compared to placebo [34]. Moreover, the safety of aprepitant against human fibroblast cells has been reported, showing the IC50 for fibroblast cells to be approximately three times higher than the IC50 for tumor cells [171].

In resumen, it seems that in addition to being potent anticancer drugs, NK-1 antagonists inherit and deploy a wide variety of favourable effects and should without doubt be considered as new, promising antineoplastic agents amongst modern anticancer strategies. The 21st century is evolving as the era of molecular targeted anticancer therapy, leading to the development of so-called “magic bullets” for anticancer therapy [171]. Rather than being “magical,” NK-1 receptor antagonists are proven to combine anticancer effects with other favourable properties. If these properties hold stand during sophisticated clinical trails, perhaps they should be considered not so much “magic” bullets, but “intelligent” bullets. In this latter, new concept of drug therapy, in one drug, a potent anticancer effect could be combined with anti-inflammatory, analgesic, anxiolytic, antidepressant, and antiemetic effects.

## 8. Future Research

The design of new therapies targeting the tumor microenvironment has been a focus of intense research over the recent years. The cellular component of the tumor microenvironment includes, among others, tumor-associated fibroblasts, endothelial and neuronal cells, and cells from the immune system.

Given the relevance of SP/NK-1 signaling in cancer, substantial efforts have been made to develop therapeutic inhibitors against NK-1 receptor. Recently, instead of its receptor, additional efforts have been made to target the NK-1 receptor ligand SP. Given the lack of chemical compounds capable of inhibiting this small peptide, the use of specific antibodies was used to address the therapeutic potential of SP inhibition in cancer. In one recent study, it was found that the use of antibodies against SP induces cell death in several cancer cell types and that this effect is accompanied by a decrease in signaling through the MAPK pathway and a decrease in the basal activity and expression of Her2 and EGFR [42]. These findings support the utility of inhibition of the SP/NK-1 signaling pathway in anticancer therapy and further suggest that indirectly targeting the NK-1 receptor via SP might be an alternative pathway for anticancer treatment. Whether targeting NK-1 via SP can overcome the caveats that limit long-term effects of NK-1 antagonists is impossible to tell, and further research must be employed. However, one must bear in mind that, as described above, the rapid turnover of NK-1 receptors on the cell surface limits long-term efficacy of NK-1 receptor antagonists [70]. Thus, blocking SP could potentially target the signal transduction of other tachykinin receptors given the ability of this peptide to bind NK-2 and NK-3 albeit with reduced affinity [29, 193]. Nevertheless, cytokine secretion mediated by activation of NK-1 receptors is also triggered by neurokinin A (NKA) [194]; moreover, it has been reported that substance K, an elongated form of neurokinin A, can induce mitogenesis [195, 196]. Hence, given the relevance of the tachykinergic system in cancer progression, the design of targeted therapies against these molecules continues to be an attractive approach for the treatment of cancer.

As mentioned above, practically no literature exists regarding the expression and function of the SP/NK-1 receptor system in CSCs. The discovery of CSCs bears enormous potential for the development of new, promising anticancer therapeutics, especially for late and recurrent cancer stages. Given the crucial role of NK-1 and SP in cancer as described in this paper, it is very likely that this receptor system bears some role either in the development or maintenance of CSCs. It is the assignment and challenge to an on-going scientific effort to identify this role.

Another promising approach in the development of alternative adjuvant tumor strategies has been the development of targeted gene therapy. The most encouraging method is that of small interfering RNA (siRNA), in which essentially any gene can be temporarily silenced for regulatory purposes. This can potentially be used for new anticancer strategies. One major limitation in the use of siRNA in vertebrates is its application. siRNA per se has unfavorable pharmacological properties and needs to be delivered intracellularly in order to develop a therapeutical effect. It was recently shown that certain, specifically designed RNA-molecules can inherit both of the above-described properties of specific gene knockdown of cancer-related targets and the simultaneous boost of the innate immune system [197, 198]. This makes these molecules especially attractive for modern anticancer strategies. For example, it is known that simultaneous knockdown of B-cell lymphoma (Bcl) 2 and RIG-I-dependent IFN-induction is superior to either therapy alone in an *in vivo* melanoma mouse model [198]. When developing similar, simultaneous anticancer strategies, NK-1 could potentially be an applicable target.

## 9. Conclusions

According to the data obtained from animal experiments, cell cultures, and clinical studies, it seems that NK-1 receptor antagonists should be effective in the treatment of affective and anxiety disorders, pain syndromes, intestinal motility disorders, and cancer. However, it must be noted that in many cases, the initial encouraging preclinical results were followed by a lack of effectiveness in human trials [199–203], and NK-1 receptor antagonists for cancer treatment have not yet been successfully translated from the bench to the bedside.

Nevertheless, as it has been extensively described in this paper, the NK-1 receptor constitutes an important network for signal transduction in the cancer microenvironment and NK-1 receptor antagonists exert three harmonizing mechanisms: first, they have an antiproliferative action due to the inhibition of tumor cell growth and induction of apoptosis; second, they inhibit angiogenesis in the tumor mass; third, they block the migration of tumor cells and thereby inhibit invasion and metastasis.

Further research and clinical trials must be carried out in order to fully reveal the beneficial effects of NK-1 antagonists in the treatment of patients suffering from cancer. Given the encouraging evidence reviewed in this paper, this task is both exciting and auspicious.
